# Thermal Decomposition Mechanism of GIS Basin Insulator and Kinetic Parameters-Based Lifetime Prediction Methodology

**DOI:** 10.3390/polym13040653

**Published:** 2021-02-22

**Authors:** Peng Ren, Qingmin Li, Honglei Liu, Yunpeng Li, Peng Peng, Naifan Xue

**Affiliations:** State Key Laboratory of Alternate Electrical Power System with Renewable Energy Sources, North China Electric Power University, Beijing 102206, China; renpieng@ncepu.edu.cn (P.R.); liuhonglei3414@ncepu.edu.cn (H.L.); lyp27@ncepu.edu.cn (Y.L.); 1182201072@ncepu.edu.cn (P.P.); NeV777@ncepu.edu.cn (N.X.)

**Keywords:** basin insulator, thermogravimetric analysis, reaction mechanism function, activation energy, lifetime expectancy

## Abstract

To reliably detect the latent defects and accurately evaluate the remaining life of gas insulated switchgear (GIS) basin insulators, more effective detection and characterization methods need to be explored. The study of pyrolysis kinetic parameters based on the intrinsic characteristics of materials provides a new way to solve this problem. First, an integral expression model of the reaction mechanism function with four parameters is proposed in this paper, which can represent various existing reaction mechanism functions with better universality and more application fields. Then, on the basis of the temperature transformation equation, an improved method for calculating the activation energy is presented, which shows higher computational accuracy than the existing methods. Further, based on a non-isothermal kinetic equation, the structure of the experimental function is given. It is a method for solving the pyrolysis reaction mechanism function of insulating materials, which can also be used to calculate the pre-exponential factor simultaneously. The thermogravimetric analysis experiment is carried out on a certain basin insulator sample at different heating rates. The pyrolysis kinetic state parameters, including the activation energy, reaction mechanism function and pre-exponential factor of the basin insulator, are calculated. Finally, the life prediction method of basin insulators is established, and the key factors affecting the life of insulators are discussed.

## 1. Introduction

Gas insulated switchgear (GIS) basin insulators are generally made by mixing epoxy resin, curing agent and filler. Bisphenol A epoxy resin is generally used with acid anhydride as curing agent. The material SiO_2_ or Al_2_O_3_ is used as filler. Due to the advantages of low curing reaction shrinkage stress, excellent mechanical and electrical properties and resistance to SF6 decomposition gas corrosion, the basin insulator has become a standard insulation component of GIS [1,2]. It can support high-voltage conductors, isolate different gas chambers and insulate from the ground. Therefore, the performance of the basin insulator has been a key factor in identifying the operation status of GIS.

Recent statistics from State Grid Corporation of China show that a series of sudden insulator flashovers, breakdowns, bursts and other GIS accidents have happened in recent years [3,4,5]. However, before applying them to the real site, all the insulators have passed factory tests, including the gas leak detection test, micro water test, AC withstand voltage test and other experimental tests, without showing any abnormality. Subsequent disassembly inspection, simulation calculation, and traceability analysis of the above faults indicated that these faults were mainly caused by the development of latent defects such as internal air gaps or cracks inside the insulator [5,6]. The epoxy resin casting material can expand or contract due to cross-linking and the temperature effect during the curing process, which easily leads to latent defects such as bubbles or air gaps in the insulator [7,8,9]. The partial discharge signal caused by internal bubbles or air gaps is weak. Meanwhile, the pressure time of a standard factory experiment or an on-site handover experiment is short, which means the faults cannot be found effectively. After a long-term operation on site, the defect gradually expands and deteriorates, which can lead to the final breakdown discharge [10,11,12,13]. At present, the insulation status diagnosis for GIS equipment mostly focuses on UHF partial discharge [14,15], dielectric properties [16], SF_6_ gas products [17], optical detection [18], vibration detection [19], etc. Through the joint efforts of several generations of researchers, these methods (UHF partial discharge, dielectric properties, SF_6_ gas products, optical detection, vibration detection, etc.) have been developed into a comprehensive detection technology. However, the combined effects of various force fields and environmental conditions usually cause the aging and deterioration of insulating materials. Sometimes the state evaluation and life prediction based on electrical characteristics under constant and uniform temperature conditions cannot provide sufficient accuracy and reliability for the fault evaluation. They still lack an effective monitoring method for the latent insulation defects of basin insulators.

On the other hand, much of the GIS equipment has been operating for more than 10 years or even longer since it was put into operation. As the service life increases, their insulation gradually deteriorates and faces maintenance, replacement or reduced-voltage operation. Therefore, it is necessary to formulate a scientific condition maintenance strategy. The statistics on the GIS interval in a certain area of the China Southern Power Grid show that most equipment has been in service for more than 15 years, and the defect rate of GIS equipment with more than fifteen-years of operation is the highest [20]. Due to the different operating environment of outdoor GIS, the deterioration degree of basin insulators with the same service life varies greatly. Therefore, it is necessary to study the general insulation life evaluation method to determine the most reasonable operation mode. At present, the evaluation research on insulation operation life only focuses on oil-paper composite insulation of transformer [21,22], cross-linked polyethylene materials of cable [23], silicone rubber [24,25], etc. The detection of basin-type insulators is mostly based on macroscopic electrical parameters such as partial discharge, and there is a lack of effective life evaluation methods.

Some eminent researchers began to use the activation energy of degradation to study the aging process of materials for different applications many years ago [26,27]. Recently, this approach has been applied to the insulating materials and shows some preliminary progress. For example, T. W. Dakin tested and analyzed insulating materials based on the Arrhenius equation [28], which predicted the aging degree of insulating materials by chemical reaction rate. In contrast, Robert R. Dixon and J. Wise obtained the aging state of insulating materials at different temperatures by extrapolation method using the Arrhenius curve [29,30]. These developments show great promise for the application of activation energy in evaluating insulation performance. In the long-term operation of GIS basin insulator, due to the coupling effects of electrical, thermal and mechanical stresses, the material can undergo complex physical and chemical changes (degradation, oxidation, crosslinking), resulting in the gradual deterioration of insulation performance. The aging and deterioration of insulators is a gradual development process. Its essence is the change of energy, which is specifically manifested with the decrease of the activation energy of the material due to the pyrolysis kinetics process. Therefore, the study of pyrolysis kinetic parameters of materials can help to reveal the development mechanism of insulation aging and deterioration. The development of thermal analysis kinetics technology provides a method for obtaining the pyrolysis kinetic parameters of materials, such as the activation energy and reaction mechanism function of materials obtained by thermogravimetric analysis, and the residual life of materials can be predicted by short-term experiments with due caution [31].

In this paper, according to the differential representation of reaction mechanism function, a general model of reaction mechanism function in integral form is constructed. In order to improve the temperature integral approximation, a more accurate calculation method of activation energy is proposed. Further, a method of solving the reaction mechanism function and pre-exponential factor based on an experimental function is given. The pyrolysis reaction activation energy, reaction mechanism function and pre-exponential factor of GIS basin insulator are solved respectively by the above methods, and the remaining life prediction equation of the basin insulator at different temperatures is proposed. Meanwhile, the influencing factors are also discussed.

## 2. Thermogravimetric Characteristics and Reaction Mechanism

### 2.1. Experimental Setup and Thermogravimetric Curves

The actual operating GIS basin insulator is taken for use as a tested object. The GIS manufacturer is entrusted, and then we get some epoxy resin composite insulation samples according to the engineering formula. The surface of the sample with alcohol is wiped, which is placed in a thermostat for 48 h to become fully dry. Then, the dried epoxy sample is ground into powder and placed in a thermostat for storage.

The TGA-DSC3+ simultaneous thermal analysis instrument from Mettler-Toledo, Zurich, Switzerland, is used to carry out the experiment under a nitrogen atmosphere of 50 mL/min, and 10 mg of each sample is placed in an alumina crucible. The heating rate is set to 5, 10, 15, 20 and 25 °C/min, respectively. The experimental temperature changes from room temperature 25 to 600 °C. Then the data of the sample mass with temperature is recorded by a computer.

**Figure 1:**
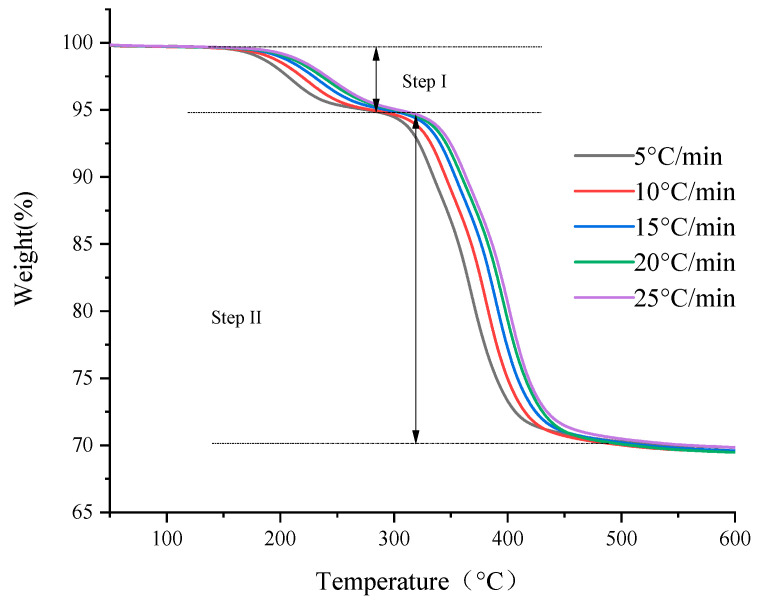
Thermogravimetric curves of the basin insulator.

**Figure 2:**
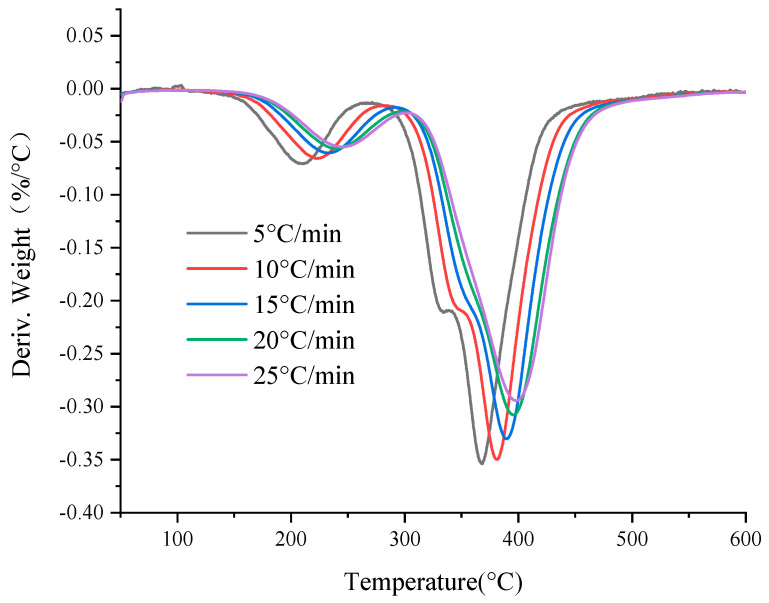
DTG curves of the basin insulator.

Figure 1 and Figure 2 show the thermogravimetric curves (TG) and first-order thermogravimetric differential curves (DTG) of the basin insulator sample at five heating rates in a nitrogen atmosphere. According to Figure 1 and Figure 2, TG curves start to show obvious mass loss between 150–180 °C and ends between 450–500 °C. Therefore, the mass loss of basin insulators mainly concentrates between 150–500 °C. From Figure 1, each TG curve experiences an inflection point in the middle part of the pyrolysis process, and thus each TG curve can be divided into two steps. It can be seen from Figure 1 and Figure 2 that with the increase of the heating rate, the initial decomposition temperature, the termination decomposition temperature and the peak temperature of the sample increase correspondingly, but the five thermogravimetric curves of the sample are roughly similar. In Figure 2, the mass loss of basin insulators is mainly at the shoulders around 330–350 °C. As the heating rate decreases, the TG curve moves toward the low temperature direction in Figure 1, which is the result of the combined effect of heat transfer resistance and mass transfer resistance. Heat transfer resistance may cause thermal hysteresis between the basin insulator sample and the heating furnace, as well as a temperature gradient inside and outside the basin insulator sample, especially at higher heating rates. The mass transfer resistance makes decomposition and vaporization not generally occur in the entire basin insulator sample, especially inside the sample, and thus the reaction shows a certain delay.

### 2.2. Thermal Decomposition Mechanism and Reaction Mechanism Function

In the process of aging and deterioration of insulating materials, there is often an induction period. This means that in the stage preceding the main process, no chemical reaction seemingly takes place [32]. In some cases, an accurate determination of the induction period is of utmost importance for safety and quality management. The induction period is often accompanied by kinetic reaction, and thus the induction period can be determined by kinetic reaction [33]. The kinetic process is often accompanied by various changes. There is the output or overflow of the material, which produces different deformations and internal stresses in the material. These changes are manifested in the solid-phase reaction forming an active area or point at the interface of the reactants, which is known as the crystal nucleus [34]. The crystal nucleus continuously grows and diffuses under the action of various stresses, which eventually leads to material failure. As a mathematical description of the growth behavior of crystal nuclei, the reaction mechanism function is used to characterize many evolutionary processes such as high-temperature pyrolysis of petroleum and coal, polymerization and solidification of polymers and dehydration and decomposition of inorganic substances [35]. It reveals the occurrence path and decomposition characteristics of chemical reactions, which can determine the stability and life of materials.

The pyrolysis kinetic equation of polymer insulating materials can be expressed by Equation (1):

(1) $$\frac{d\alpha }{dT}=\left(\frac{A}{\beta }\right)\exp\left(-\frac{E}{RT}\right)f\left(\alpha \right)$$

where *α* is the conversion rate, *A* is the pre-exponential factor (frequency factor), *T* is the absolute temperature, *E* is the activation energy, *R* is the ideal gas constant, and *β* = *dT*/*dt* is the heating rate. *f*(*α*) is the reaction mechanism function, which characterizes a certain functional relationship between the reaction rate and the conversion rate *α*. *G*(*α*) is the integral form of *f*(*α*), which can be expressed by Equation (2):

(2) $$G\left(\alpha \right)=\int_{0}^{\alpha }\frac{d\alpha }{f\left(\alpha \right)}$$

In the solid-phase reaction, the reaction mechanism function can be used to describe a specific reaction type, which mathematically converts it into a rate equation. Based on mechanism assumptions, mechanism functions are usually divided into nucleation, geometric contraction, diffusion and series reaction. Some commonly used mathematical expressions are shown in Table 1 [36,37].

These reaction mechanism function models give a basic description of the pyrolysis process of many solid materials. Due to the complexity of the pyrolysis reaction itself, the irregularity of the actual material particle structure and shape, the irregularity of the sample accumulation in the crucible and the variability of the physical and chemical properties of the reactants [38,39], it is often found that the actual thermal decomposition curve does not match the predicted reaction mechanism [40,41]. The solved reaction mechanism function can inevitably be distorted. For a long time, due to the lack of a more general characterization of the reaction mechanism function, thermal analysis of materials has been puzzling and complex.

Aiming at the differential representation form of the reaction mechanism function, Sestak and Berggren proposed a more suitable empirical model by analyzing the above four types of reaction mechanism functions, as shown in Equation (3) [42]:

(3) $$f\left(\alpha \right)=\alpha^{m}{\left(1-\alpha \right)}^{n}{\left(-\ln(1-\alpha )\right)}^{p}$$

where *m*, *n* and *p* represent the exponential factors of different values, and one of them is always equal to 0 in the calculation. In general, the calculation of the remaining life of the material is closely related to the integral expression form G(*α*) of the reaction mechanism function [43]. Therefore, it is important to study the integral expression model followed by the pyrolysis reaction of materials. Based on Equation (3), this paper proposes a four-parameter general characterization of the reaction mechanism function in integral form:

(4) $$G\left(\alpha \right)=q\alpha^{m}{\left(1-\alpha \right)}^{n}{\left(-\ln(1-\alpha )\right)}^{p}$$

**Figure 3:**
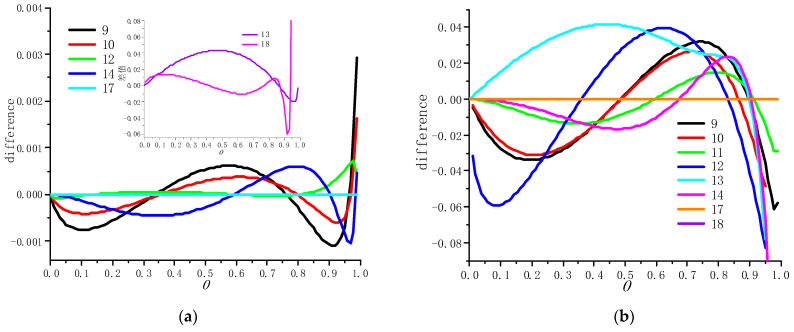
The difference between (**a**) Equation (4), (**b**) Equation (3) and the existing reaction mechanism function.

For specific material pyrolysis reactions, *q*, *m*, *n* and *p* are constants. In order to verify the universality and accuracy of Equation (4), the difference between Equation (4) and the integral form of the existing reaction mechanism functions in Table 1 is taken as the evaluation index. The calculated results are compared with those solved by Equation (3), as shown in Figure 3.

It can be seen from Figure 3 that Equation (4) can characterize the existing reaction mechanism functions in Table 1 as long as appropriate parameter values of *q*, *m*, *n* and *p* are selected. Compared with Equation (3), Equation (4) shows higher accuracy and applicability in the whole conversion range between 0 and 1.

## 3. An Improved Solution Method for Thermal Kinetic Parameters

### 3.1. An Improved Method of Temperature Integral Analysis

The methods to solve pyrolysis kinetic parameters from reaction kinetic equations are mainly divided into integral type and differential type. Compared with the differential method which is more susceptible to experimental data noise [44], the integral method is widely used in thermal analysis kinetics. However, due to the Arrhenius temperature integration problem, researchers from different countries have tried to transform temperature integration into several finite series or rational function approximations to improve accuracy. The accuracy of these approximate equations is low, and many works often ignore the accuracy and value range of the approximate equations when using integral method to deal with the thermogravimetric data, leading to contradictory conclusions or wrong dynamic parameters.

Separate the variables of Equation (1) and make an integration of the both sides, then Equation (5) is obtained:

(5) $$G(\alpha )=\int_{T_{0}}^{T}\frac{A}{\beta }\exp(-\frac{E}{RT})dT\approx \int_{0}^{T}\frac{A}{\beta }\exp(-\frac{E}{RT})dT=\frac{AE}{\beta R}P(u)$$

*P (u)* is called the Arrhenius temperature integral, which is shown in Equation (6), where *u* = *E*/*RT*.

(6) $$P(u)=\int_{\infty }^{u}-\frac{e^{-u}}{u^{2}}du$$

By the stepwise integral expansion of the variable *u*, the following expression is obtained:

(7) $$\begin{array}{l} P(u)=\int_{\infty }^{u}-(\frac{e^{-u}}{u^{2}})du=\int_{\infty }^{u}1/u^{2}{\mathrm{de}}^{-u}\\ =\frac{e^{-u}}{u^{2}}{|}_{\infty }^{u}-\;\int_{\infty }^{u}e^{-u}du^{-2}\\ =\frac{e^{-u}}{u^{2}}-\int_{\infty }^{u}e^{-u}(-2)u^{-3}du\\ =\frac{e^{-u}}{u^{2}}-\frac{2e^{-u}}{u^{3}}+\frac{6e^{-u}}{u^{4}}-\int_{\infty }^{u}24u^{-5}{\mathrm{de}}^{-u}\\ =\frac{e^{-u}}{u^{2}}(1-\frac{2!}{u}+\frac{3!}{u^{2}}-\frac{4!}{u^{3}}+\cdots )\\ =\frac{e^{-u}}{u^{2}}H\left(u\right) \end{array}$$

Based on the stepwise integration, appropriate interception of the expansion equation is carried out through various mathematical treatments. Some researchers have successively given several approximations, such as the Doyle approximation [45], Starink approximation [46,47], Madhusudanan-Krishnan-Ninan (MKN) approximation [48,49], etc. Among them, Starink approximation includes two types and MKN approximation includes three types, as shown in Table 2.

The approximate equation of exponential form in Table 2 can actually be expressed by the unified undetermined coefficient in Equation (8):

(8) $$P(u)=\frac{\exp(au+b)}{u^{m}}$$

From Equation (7), the following can be obtained:

(9) $$H(u)=\frac{P(u)}{e^{-u}/u^{2}}=1-\frac{2!}{u}+\frac{3!}{u^{2}}-\frac{4!}{u^{3}}+\cdots$$

Substitute Equation (9) into Equation (8), then Equation (10) is shown:

(10) $$H(u)=\frac{\exp[(a+1)u+b]}{u^{m-2}}$$

Take the logarithm of Equation (10) to get:

(11) ln[H(u)]=(2−m)lnu+(1+a)u+b

Further differentiate the two sides of Equation (11) to obtain:

(12) $$\frac{H^{\prime }(u)}{H(u)}=\frac{2-m}{u}+(1+a)$$

The derivation of Equation (9) is shown by Equation (13):

(13) $$H^{\prime }(u)=H(u)(1+\frac{2}{u})+P^{\prime }(u)e^{u}u^{2}$$

The derivation of Equation (7) is shown by Equation (14):

(14) $$P^{\prime }(u)=-e^{-u}/u^{2}$$

Substitute Equation (14) into Equation (13), we can obtain:

(15) $$H^{\prime }(u)=(1+\frac{2}{u})H(u)-1$$

Combine Equation (12) with Equation (15), we can obtain:

(16) $$\frac{u}{H(u)}=m-au$$

Since *u* is the independent variable and *u*/*H (u)* solved by Simpson is the dependent variable, the undetermined parameters *m* = 1.868479 and *a* = −1.001749 can be obtained from Equation (16) by linear fitting. For most solid-phase reactions, the value *u* is between 15 and 60 [47]. By substituting the values *m* and *a* into Equation (8), it can be obtained that the average value *b* in the expanded range of *u* from 5 to 70 is −0.458584. Therefore, this paper obtains a transform form of temperature integral, as shown in Equation (17).

(17) $$P(u)=\frac{\exp\left(-1.001749u-0.458584\right)}{u^{1.868479}}$$

**Figure 4:**
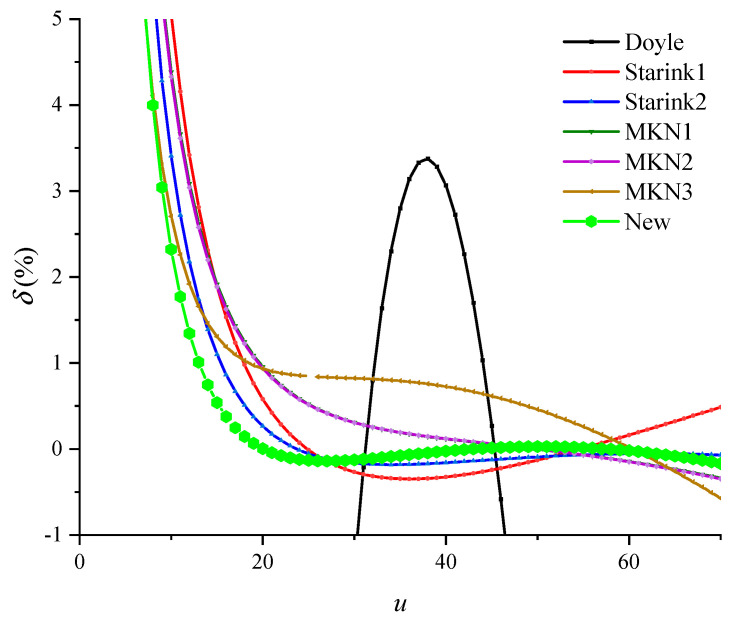
Percent deviation of the temperature integral approximation.

In order to evaluate the accuracy of Equation (17), let *u* take a value between 5 ≤ *u* ≤ 70. The Simpson’s numerical solution of temperature integral is taken as the exact value, which is compared with other exponential approximations in Table 2. The percentage deviation is shown in Figure 4, and the value range of *u* at typical percentage deviation is shown in Table 3.

It can be seen from Figure 4 and Table 3 that the Doyle approximation is not accurate in the entire range of *u*, which should be used with caution in the calculation of activation energy. However, the accuracy of the new temperature integral transformation equation is much better than that of other exponential integral approximations in most of the value range of *u*. Moreover, the integral transformation equation is obtained based on strict mathematical derivation, and thus the result is more reliable.

### 3.2. An Improved Algorithm for Solving Activation Energy

When applying the Flynn-Wall-Ozawa method to solve the activation energy, it is unnecessary to set the reaction mechanism function [50,51], which is used to avoid some calculation errors. However, the Flynn-Wall-Ozawa method uses the Doyle temperature integral approximation in the derivation, which may bring new calculation errors. For this reason, the temperature integral transformation equation proposed in this paper can be incorporated into the Flynn-Wall-Ozawa method to improve the calculation accuracy of activation energy.

Take the logarithm of both sides of Equation (5) to get:

(18) $$\ln G(\alpha )=\ln\frac{AE}{\beta R}+\ln P(u)$$

Substitute Equation (17) into Equation (18), the following expression is obtained:

(19) $$\ln\frac{\beta }{T^{1.868479}}=[\ln\frac{AE}{G(\alpha )R}-0.458584-1.868479\ln\frac{E}{R}]-1.001749\frac{E}{RT}$$

For the thermogravimetric curves obtained at different heating rates, the first term on the right side of Equation (19) is a constant when the conversion rates are the same. By doing a function diagram of ln(*β*/*T*^1.868479^) and 1/*T*, the slope of the function is obtained as −1.001749 *E*/*R*, and the activation energy can be calculated by the slope.

In order to evaluate the calculation accuracy of the improved activation energy algorithm, Equation (8) is substituted into Equation (18) to obtain:

(20) $$\ln G(\alpha )=b+\ln(\frac{AE_{1}}{\beta R})+au_{1}-m\ln u_{1}$$

where *E*_1_ represents the activation energy approximately solved by the exponential integral. *u*_1_ is equal to *E*_1_/(*RT*).

Differentiate both ends of Equations (20) and (21) is obtained:

(21) $$\frac{\mathrm{dln}[G(\alpha )]}{d(1/T)}=\frac{E_{1}}{R}(a-\frac{m}{u_{1}})$$

Differentiate both ends of Equation (18) and we can obtain:

(22) $$\frac{\mathrm{dln}[G(\alpha )]}{d(1/T)}=\frac{E}{R}\frac{d\ln[P(u)]}{du}$$

Since the activation energy represented by Equation (22) does not adopt integral approximation, its solution is the real activation energy *E*. Therefore, the relative error of activation energy can be defined by Equation (23):

(23) $$\delta =\frac{E_{1}-E}{E}\times 100\%$$

Considering the Equations (21)–(23), the following can be obtained:

(24) $$\delta =\frac{1}{a}\{\frac{d\ln[P(u)]}{du}+\frac{m}{u}\}-1$$

**Figure 5:**
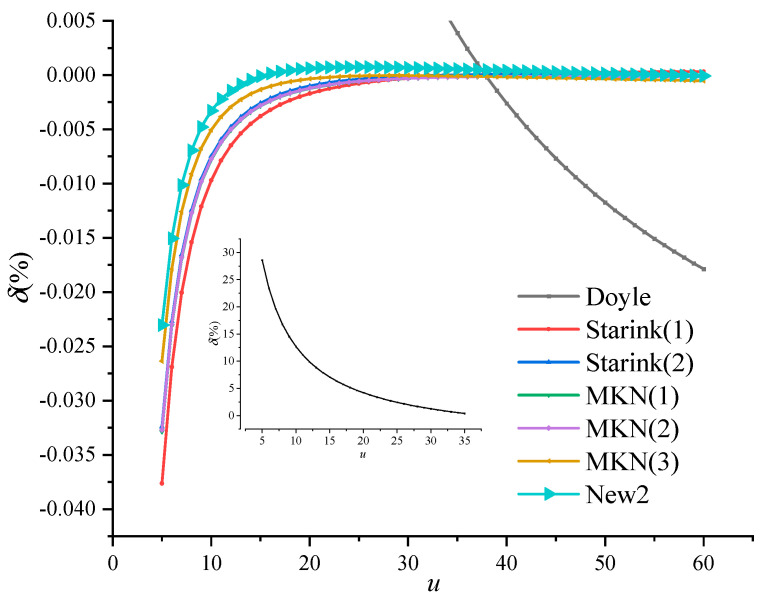
Percent deviation of activation energy of the temperature integral transformation.

From Equation (24), the activation energy errors of various exponential approximations can be obtained, as shown in Figure 5. It can be seen that the activation energy calculated by the Flynn-Wall-Ozawa method improved by the integral transformation shows the highest accuracy.

From step one of the basin insulator thermogravimetric curve, the conversion rate *α* is selected to be between 0.2 and 0.8 with an interval of 0.1. At the same conversion rate, different heating rates are substituted into Equation (19), and the average activation energy corresponding to different conversion rates is 86 (± 4) kJ/mol. By repeating the above steps based on the improved activation energy calculation method, the average activation energy of the reaction step II of the basin insulator is 171 (± 6) kJ/mol. If the average of the sum of the activation energies, including reaction step one and reaction step two, is defined as the average activation energy of the total reaction, then the average activation energy value of the total reaction is 129 (±5) kJ/mol.

### 3.3. Solution Method of Reaction Mechanism Function

If *α* = 0.5 is taken as the reference point, the following can be obtained from Equation (5):

(25) $$G(0.5)=(\frac{AE}{\beta R})P(u_{0.5})$$

where *u*_0.5_ is the corresponding value at *α* = 0.5. Divide Equations (25) and (10) to get:

(26) $$\frac{P(u)}{P(u_{0.5})}=\frac{G(\alpha )}{G(0.5)}$$

Substitute Equations (4) and (17) into Equation (26) and we can obtain:

(27) $$\frac{\frac{\exp\left(-1.001749u-0.458584\right)}{u^{1.868479}}}{\frac{\exp\left(-1.001749u_{0.5}-0.458584\right)}{u_{0.5}{}^{1.868479}}}=\frac{\alpha^{m}{\left(1-\alpha \right)}^{n}{\left(-\ln(1-\alpha )\right)}^{p}}{0.5^{m}{\left(1-0.5\right)}^{n}{\left(-\ln(1-0.5)\right)}^{p}}$$

The activation energy *E* and temperature *T* obtained in the range of conversion *α* of polymer insulating materials can be substituted into the left side of Equation (27) to form the experimental function curve. If the pyrolytic reaction of the material can be described by a certain type of reaction mechanism function, the experimental curves should coincide with each other regardless of the heating rate. Substitute different conversion rates *α* into the right side of Equation (27), and fit the experimental curve obtained by Equation (12) by the least square method to obtain the corresponding parameters *m*, *n* and *p*.

Substitute Equations (4) and (17) into Equation (18), and the following expression is obtained:

(28) $$q\alpha^{m}{\left(1-\alpha \right)}^{n}{\left(-\ln(1-\alpha )\right)}^{p}=\frac{AE}{\beta R}\frac{\exp\left(-1.001749u-0.458584\right)}{u^{1.868479}}$$

Since the parameters *m*, *n*, *p*, *u*, *E*, *β* and *R* in Equation (28) are known, the unknown ones only include the values of *q* and *A*. If the parameters corresponding to any two conversion rates *α* are substituted into Equation (28), the values of *q* and *A* can be obtained by solving the equations.

**Figure 6:**
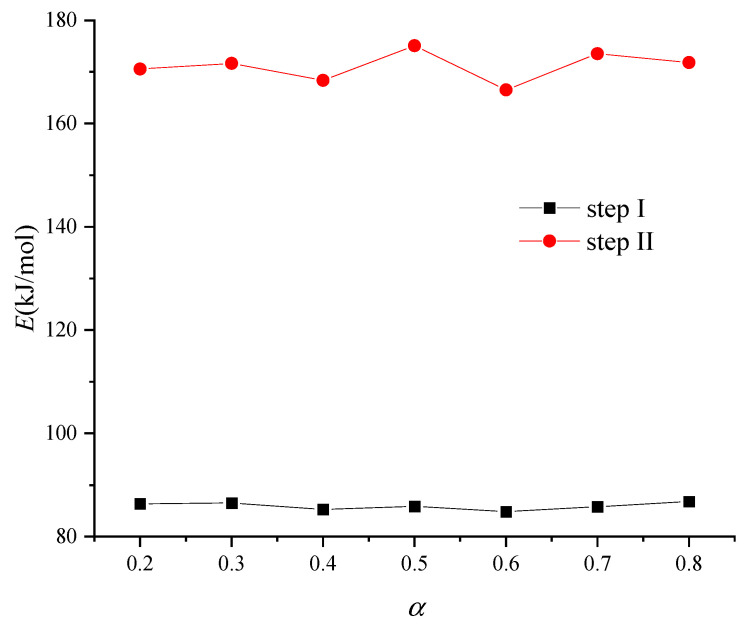
Relationship between activation energy and conversion rate.

The complexity of the thermal decomposition reaction can be judged from the relationship between conversion rate and activation energy [51]. Figure 6 shows the relationship curve between activation energy and conversion rate. It can be seen from the figure that the activation energy values of step I and step II hardly change with the conversion rate *α*, which indicates that the reaction process follows a single reaction mechanism function.

**Figure 7:**
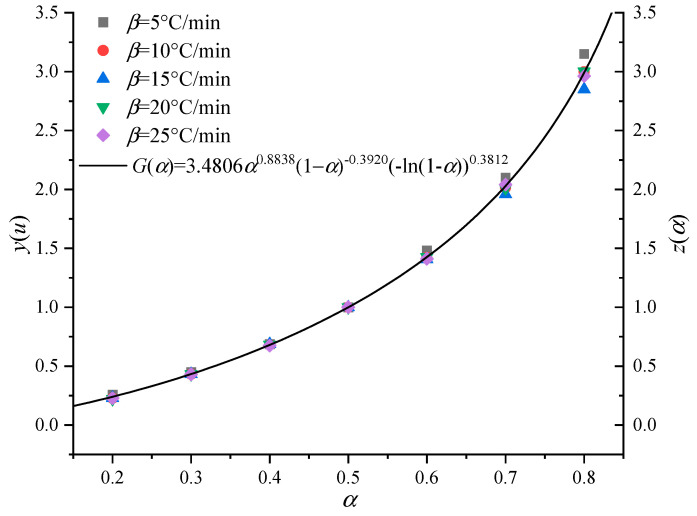
Experimental curves of the basin insulator stage I.

**Figure 8:**
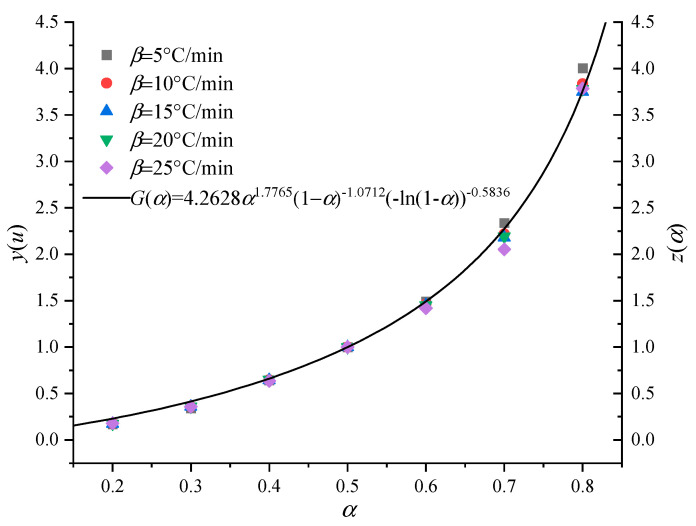
Experimental curves of the basin insulator stage II.

Figure 7 and Figure 8 are the experimental function curves of basin insulators at five heating rates. It can be seen that almost all the experimental curves overlap at different temperature heating rates, which indicates that step I and step II of the pyrolysis reaction of the basin insulator can be characterized by a single reaction mechanism function. According to the aforementioned solution method, their respective reaction mechanism functions can be obtained: *G*(*α*) = 3.4806*α*^0.8838^ (1 − *α*)^−0.3920^ (−ln(1 − α))^0.3812^ and *G*(*α*) = 4.2628α^1.7765^ (1 − α)^−1.0712^ (−ln(1 − α))^−0.5836^. Previous studies have shown that the phase interface reaction in the solid phase reaction process can be hindered in some way and result in the fluctuation of activation energy, which makes the kinetic index appear as a fraction or a decimal. Although the physical meaning is not very clear, it can better describe some reactions and effectively characterize the non-idealization degree of the basin insulator pyrolysis reaction. At the same time, the average values of the pre-exponential factor *A* under-five heating rates are 4.80 × 10^8^ and 4.74 × 10^13^ s^−1^.

## 4. Life Assessment

In essence, the life problem is the rate of material chemical reaction. The insulation material can degrade and crack under the action of external factors such as light, temperature and humidity. The speed of its rate can determine the service life of the insulating material. The reaction rate problem is a kinetic problem, which can be expressed by kinetic parameters. Thus, the kinetic parameters are related to the life of the material.

### 4.1. Life Calculation

Substitute *β* = *dT*/*dt* into Equation (1) to get:

(29) $$\frac{d\alpha }{dt}=A\exp(-\frac{E}{RT})f\left(\alpha \right)$$

By transforming and integrating Equation (29), the following expression is obtained:

(30) $$\int_{0}^{\alpha }\frac{d\alpha }{f\left(\alpha \right)}=G\left(\alpha \right)=A\exp(-\frac{E}{RT})t=k\left(T\right)t$$

where *k*(*T*) = *A*exp(−*E*/*RT*) is the famous Arrhenius equation. Equation (30) can be further transformed into:

(31) $$t=\frac{G\left(\alpha \right)}{k\left(T\right)}$$

It can be seen from Equation (31) that the problem of the service life of a material at a certain temperature is solving a certain reaction mechanism function *G*(*α*) caused by material degradation or crack and the reaction rate constant *k*(*T*) at a certain temperature. When the conversion rate of the polymer reaches 5%, the life of the material can be considered at its end [52,53], and has been the criterion to evaluate the end of life. Considering the thermal weight loss of the basin insulator as a total reaction, the reaction with a conversion rate of 5% is mainly concentrated on step one. Thus, the remaining life of the basin insulator is mainly determined by the reaction mechanism function of step one, which corresponds to the conversion rate of about 32% in step one. Since the aging of basin insulators is a very slow process under actual operating conditions and its chemical reaction rate is the result of the joint action of many factors, the average activation energy is used to calculate its reaction rate. The reaction mechanism function and the chemical reaction rate constants at different temperatures are substituted into Equation (31) to obtain the life of insulators at different temperatures, as shown in Table 4.

It can be seen from Table 4 that as the operating temperature increases, the service life of the basin insulator gradually decreases. When the operating temperature of the material is 80 ℃, the remaining life of the basin insulator is about 175 years. When the operating temperature of the material reaches the limit temperature of 105 °C specified in IEC62271-1-2017, the life of the insulator is only about 10 years. Therefore, temperature shows a great influence on the life of the GIS basin insulator.

### 4.2. Discussion

Much research has been conducted on the service life of epoxy resin composite materials. Peter H. F. conducted electrical aging experiments on epoxy resin composite materials at room temperature and obtained the epoxy resin life of 37 years by fitting the V-t curve [54]. M. Joy Thomas used the Weibull distribution to study the life of epoxy resin composites filled with nano-Al_2_O_3_, and the life was about 8.6 years under a temperature of 60 °C and an electric field strength of 3 kV/mm [55]. Reference [56] studied the thermal deformation temperature of epoxy resin by isothermal annealing method, and its service life is about 20 years at 115 °C. It should be noted that, due to the different types of base materials, curing agents with fillers used in different types of epoxy resins, and differences in curing processes, the expected life expectancies are usually different. The lifetime of the basin insulator predicted by the proposed method in this paper is in good agreement with that of the epoxy resin obtained by other researchers. Compared with other methods, the life prediction method based on the reaction mechanism function proposed in this paper can obtain the three dynamic factors of basin insulators through rapid experiments, which can further predict the remaining life of GIS insulators through the intrinsic properties of materials. On the one hand, this method can help power grid companies to select insulators produced by different manufacturers. On the other hand, it can also be used to predict the service life of GIS insulators in operation, which can help arrange a reasonable maintenance plan.

## 5. Conclusions

(1) The temperature heating rate shows great influence on the thermogravimetric curve of GIS basin insulators. As the heating rate decreases, the TG curve of the GIS insulator shifts towards the low temperature. The corresponding initial temperature, termination temperature, maximum mass loss temperature and peak temperature also decrease accordingly.
(2) Based on the differential expression of the reaction mechanism function, a universal integral expression of the reaction mechanism function is proposed, which shows better applicability and accuracy in a larger conversion rate range.
(3) Based on the approximate treatment of temperature integral, the Arrhenius temperature integral transformation form is proposed, which shows better applicability and accuracy in the whole value range of *u*. On this basis, an improved calculation method of activation energy is presented, which can promote higher accuracy of the solution.
(4) According to the pyrolysis kinetic equation of the material, a method for solving the pyrolysis reaction mechanism function of the insulating material is given. Based on this method, the pre-exponential factor can be obtained at the same time.
(5) Based on the obtained activation energy, reaction mechanism function and pre-exponential factor, a residual life prediction method of GIS basin insulators using the pyrolysis kinetic state parameters is proposed. Further analysis shows that the service life of basin insulators is most affected by the operating temperature.

## Figures and Tables

**Table 1:** The commonly used kinetic model of heterogeneous reaction.

No	Model	Differential Form *f* (*α*) = 1/*k dα/dt*	Integral Form *G (α)* = *kt*
Nucleation models			
1	Avrami-Erofeev, *m* = 4 (S shape)	4(1 − *α*)(−ln(1 − *α*))^3/4^	(−ln(1 − *α*))^1/4^
2	Avrami-Erofeev, *m* = 3 (S shape)	3(1 − *α*)(−ln(1 − *α)*)^2/3^	(−ln(1 − *α*))^1/3^
3	Avrami-Erofeev, *m* = 2 (S shape)	2(1 − *α*)(−ln(1 − *α*))^1/2^	(−ln(1 − *α*))^1/2^
4	Avrami-Erofeev, *m* = 1.5 (S shape)	3/2(1 − *α*)(−ln(1 − *α*))^1/3^	(−ln(1 − *α*))^2/3^
5	Power law, *n* = 1/4 (Acceleration model)	4*α*^3/4^	*α* ^1/4^
6	Power law, *n* = 1/3 (Acceleration model)	3*α*^2/3^	*α* ^1/3^
7	Power law, *n* = 1/2 (Acceleration model)	2*α*^1/2^	*α* ^1/2^
8	Power law, *n* = 3/2 (Acceleration model)	2/3*α*^−1/2^	*α* ^3/2^
Geometrical contraction models			
9	Phase boundary, *n* = 2 (Deceleration model)	2 (1 − *α*)^1/2^	1 − (1 − *α*)^1/2^
10	Phase boundary, *n* = 3 (Deceleration model)	(1 − *α*)^2/3^	1 − (1 − *α)*^1/3^
Diffusion models			
11	One-dimensional diffusion (Deceleration model)	*α* ^−1^	1/2*α^2^*
12	Two-dimensional diffusion (Deceleration model)	2(1 − *α*)^1/2^(1 − (1 − *α*)^1/2^)^1/2^	1/2(1 − (1 − *α*)^1/2^)^1/2^
13	Three-dimensional diffusion (Deceleration model)	3/2((1 − *α*)^1/2^ − 1)^−1^	1−2/3*α* − (1− *α*)^1/2^
14	Jader’s type diffusion (Deceleration model)	3/2(1 − *α*)^2/3^((1 − *α*)^1/3^ − 1)^−1^	(1 − (1 − *α*)^1/3^)^2^
Reaction-order models			
15	Phase boundary, *n* = 1 (Linear model)	1	*α*
16	1st order(Deceleration model)	1 − *α*	−ln(1 − *α*)
17	2nd order (Deceleration model)	(1 − *α*)^2^	(1 − *α*)^−1^ − 1
18	3rd order (Deceleration model)	(1 − *α*)^3^	1/2((1 − *α*)^−2^ − 1)

**Table 2:** Temperature integral approximation in exponential form.

Approximate Name	Expression
Doyle	P(u)=exp(−5.3308−1.0516u)
Starink	P(u)=exp(−0.235−1.95lnu−u)(I) P(u)=exp(−0.312−1.92lnu−1.0008u)(II)
MKN	P(u)=exp(−0.297580−1.921503lnu−1.000953u)(I) P(u)=exp(−0.299963−1.920620lnu−1.000974u)(II) P(u)=exp(−0.389677−1.884318lnu−1.001928u)(III)

**Table 3:** The value range of *u* for different temperature integral equation at typical percentage deviation.

Approximate Name	Typical Percentage Deviation/%		
	*δ* < 0.1	*δ* < 0.5	*δ* < 1.0
Doyle	—	*u* = 31,45	31 ≤ *u* ≤ 32, 45 ≤ *u* ≤46
Starink 1	24 ≤ *u* ≤ 26,52 ≤ *u* ≤ 58	21 ≤ *u* ≤ 66	≥18
Starink 2	≥49	≥19	≥16
MKN l	42 ≤ *u* ≤ 57	≥22	≥20
MKN 2	42 ≤ *u* ≤ 56	≥26	≥20
MKN 3	59 ≤ *u* ≤ 62	49 ≤ *u* ≤ 68	≥19
New	19 ≤ *u* ≤ 66	≥16	≥14

**Table 4:** Service life of the basin insulator at different temperatures.

*T*/℃	*k*_T_	Service Life/Year
50	9.34 × 10^−13^	9819.88
60	3.88 × 10^−12^	2366.80
70	1.49 × 10^−11^	619.47
80	5.25 × 10^−11^	174.85
90	1.73 × 10^−10^	52.80
100	5.38 × 10^−10^	17.05
105	9.26 × 10^−10^	9.90
110	1.57 × 10^−9^	5.83
120	4.36 × 10^−9^	2.11

## Data Availability

The data presented in this study are available by contacting the corresponding author.
